# NMR-Based Metabolomic Analyses to Identify the Effect of Harvesting Frequencies on the Leaf Metabolite Profile of a *Moringa oleifera* Cultivar Grown in an Open Hydroponic System

**DOI:** 10.3390/molecules26082298

**Published:** 2021-04-15

**Authors:** Lavhelesani R. Managa, Elsa S. du Toit, Gerhard Prinsloo

**Affiliations:** 1 Department of Plant and Soil Sciences, Faculty of Natural and Agricultural Sciences, University of Pretoria, Private Bag X20, Hatfield, Pretoria 0028, South Africa; 2 Africa Institute of South Africa (AISA), Human Sciences Research Council (HSRC), Pretoria 0002, South Africa; 3 Department of Agriculture and Animal Health, Science Campus, University of South Africa, Florida, Johannesburg 1710, South Africa; prinsg@unisa.ac.za

**Keywords:** *Moringa oleifera*, metabolites, hydroponic, metabolomics, medicinal plant, harvesting, gamma-aminobutyric acid, chlorogenic acid, ferulic acid, vanillic acid

## Abstract

*Moringa oleifera* Lam. is one of the world’s most useful medicinal plants. Different parts of the *M. oleifera* tree contain a rich profile of important minerals, proteins, vitamins, and various important bioactive compounds. However, there are differences in the phytochemical composition of the medicinal plant’s raw materials due to seasonal variation, cultivation practices, and post-harvest processing. The main objective of this study was therefore to determine the effect of harvesting frequencies on selected bioactive compounds of a *M. oleifera* cultivar (PKM1) grown in a hydroponic system under a shade net structure. Three harvesting frequency treatments were applied in the study, with the plants harvested at every 30 days (high frequency), 60 days (intermediate frequency), and 90 days (low frequency) respectively. ^1^H-NMR was used for data acquisition, and multivariate data analysis by means of principal component analysis (PCA), partial least square discriminatory analysis (PLS-DA), and orthogonal partial least square discriminatory analysis (OPLS-DA) were applied to determine the changes in the leaf metabolite profile, and also to identify the spectral features contributing to the separation of samples. Targeted metabolite analysis was used to match the NMR peaks of the compounds with the NMR chemical shifts of the contribution plot. The contribution plot showed that the increase in concentration of some compounds in aliphatic, sugar and aromatic regions contributed to the separation of the samples. The results revealed that intermediate and low harvesting frequencies resulted in a change in the leaf metabolite profile. Compounds such as chlorogenic acid, ferulic acid, vanillic acid, wogonin, esculetin, niazirin, and gamma-aminobutyric acid (GABA) showed an increase under intermediate and low harvesting frequencies. These results provide insight into the effect of harvesting frequencies on the metabolite profile and associated medicinal activity of *M. oleifera*.

## 1. Introduction

The demand for *Moringa oleifera* Lam. leaf material is on the rise in many parts of the world, especially in Africa, India and Philippines notably. This is due to the growing interest in the usage of Moringa tree for herbal medicine, and in development of other pharmaceutical products. Different parts of the Moringa tree contain a rich profile of important minerals, proteins, vitamins, β carotene, amino acids, and various phenolics in addition to a rich and rare combination of zeatin with several flavonoid pigments [1,2]. Various forms of *M. oleifera* leaf extracts proved to exhibit various therapeutic activities such as anti-cancer, anti-hypertension, anti-diabetes, anti-epilepsy, and many more [3,4,5,6,7]. Of particular note is that extracts of the leaves were found to induce apoptosis (the process of programmed cell death) in the human body when ingested orally [8], suggesting that *M. oleifera* could be a potential valuable alternative in the treatment of cancer. These remarkable anti-cancer properties could be attributed to a wide array of bioactive compounds, specifically eugenol, isopropyl isothiocynate, d-allose, niazimicin, niazirin, β-sitosterol-3-*O*-β-d-glucopyranoside, and hexadeconoic acid ethyl ester [4,7,9]. *Moringa oleifera* leaf extract was also reported to possess strong anti-oxidant activity due to its high polyphenols, flavonoids, flavonols, and vitamin C content [10,11,12]. Moreover, the fact that the aqueous leaf extract of *M. oleifera* has proven to be safe when administered orally [13] led to an increase in demand for its derivatives. 

However, change in the metabolites profile is one of the major limitations in using plant materials as sources of various medicinal products. There could be differences in the chemical composition of medicinal and aromatic plant (MAP) raw materials due to natural variation, cultivation practices, and post-harvest processing [14]. In addition, secondary metabolites are also synthesized in specialized cell types and only during particular growth stage, or under specific conditions, thus making their extraction irregular [15,16,17]. Moringa species in particular, are very rich in useful bioactive compounds, but their chemical composition has been shown to be greatly influenced by biotic and abiotic factors [2,18,19]. The chemical composition of a plant is crucial to retain its activity for the use as food supplement and in treatment of diseases. Moreover, moringa is a tree that is usually harvested several times within the season, and changes in the metabolites profile may occur due to harvesting frequencies as well as post-harvest treatment [20,21]. However, the effect of harvesting frequencies on the leaf metabolites profile of *M. oleifera* have not been reported prior to this study. 

In addition, despite the recent advances in plant metabolomics research, only a few studies have been reported on the application of metabolomics for *M. oleifera* cultivars. Using techniques such as LC-MS, GC-MS, and NMR spectroscopies, metabolomics provides a rapid and high throughput method to efficiently detect the changes in the metabolic profile of plant materials. Thus, the effect of various biotic and abiotic factors on the accumulation of secondary metabolites could be efficiently determined with metabolomics technology. ^1^H-NMR spectroscopy is particularly a good choice in plant metabolomics studies given the universal occurrence of protons in organic metabolites, and can be successfully applied in moringa plant research [22,23,24]. It enables analyses of complex samples such as herbs and phytomedicines without separation but with good accuracy and consistency based on the entire chemical composition of the samples. In principle, ^1^H-NMR provides a reliable profile of each raw material sample, and quantities are reflected in the integrals of the individual signals of the spectrum [24,25].

Against this background, the main objective of this study is to determine the effect of harvesting frequencies on the leaf metabolites profile of a *M. oleifera* cultivar (PKM1) grown in an open hydroponic system in a shade net structure. The study utilized ^1^H-NMR-based metabolomics and chemometric analysis to determine the changes in the plant metabolite profile. 

## 2. Results and Discussion

In the current study, untargeted and targeted NMR-based metabolomics was applied to detect the changes in the leaf metabolite profile of the *M. oleifera* cultivar due to harvesting frequencies, and associated conditions in the open hydroponic system. The obtained spectral data were then used for biochemical interpretation of samples. As indicated in Table 1, the number of samples analyzed varied from month to month based on the harvesting frequency for each month. In total, there were nineteen (19) extract samples for high harvesting frequency group, ten (10) for intermediate harvesting group, and eight (8) for low harvesting frequency group. Samples from June to September were excluded from metabolomics analysis as the leaf quality was affected by winter season. Moringa leaf productivity could be severely affected in areas with very low temperature and freezing winter temperatures like in the current study area. In addition, the outliers were removed before analysis of the data commenced using the Hotelling’s T2 range and Distance to Model (DMod) tests in SIMCA.

### 2.1. Changes in the Metabolite Profile

**Figure 1:**
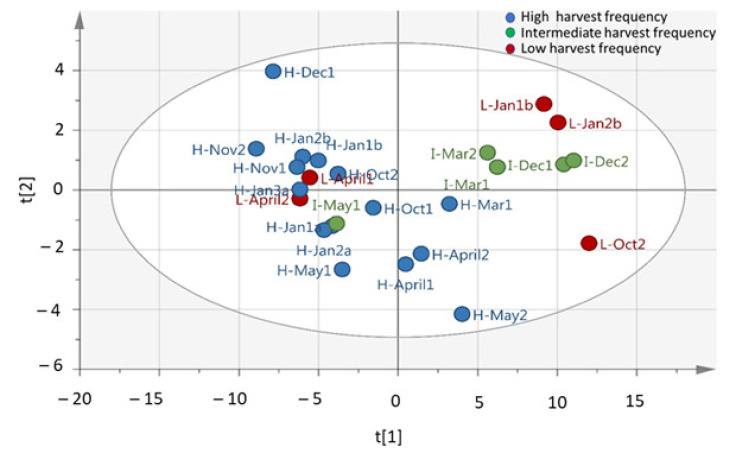
Principal component analysis (PCA) analysis (PC1 and PC2) of *M. oleifera* (cultivar PKM1) metabolite profile under high, intermediate, and low harvesting frequencies (R^2^X = 0.94; Q^2^ = 0.844). R^2^X and Q^2^ represent the cumulative values of all the components of the model. Low harvest frequency (red dots), intermediate harvest frequency (green dots), high harvest frequency (blue dots). H-30 days harvesting frequency; I-60 days harvesting frequency; L-90 days harvesting frequency; Jan-a = January, A = April, Mar = March, May = May, Oct = October, Nov = November, Dec = December; Jan-b–January of the next year. R^2^X[1] = 0.78; R^2^X[2] = 0.058.

**Figure 2:**
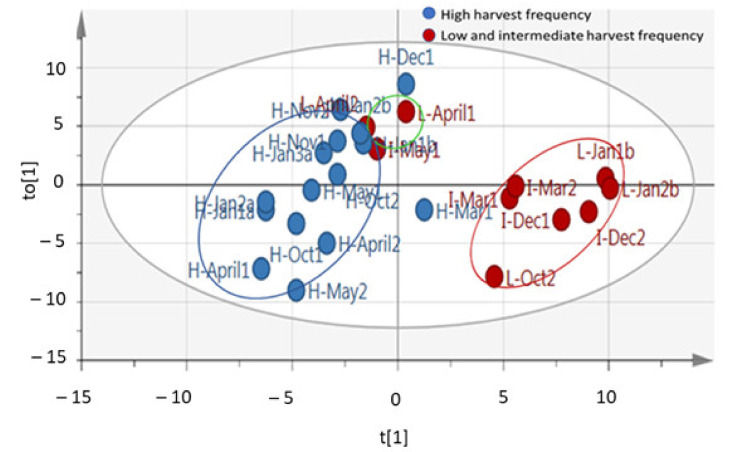
Orthogonal partial least square discriminatory analysis (OPLS-DA) score plot showing the predictive (t[1]) and orthogonal (to [1]) components of ^1^H-NMR spectral data of *M. oleifera* (cultivar PKM1) metabolites profile under low/intermediate and high harvesting frequencies, showing three distinct clusters (R^2^X = 0.832; R^2^Y = 0.643; Q^2^ = 0.371). R^2^X, R^2^Y, and Q^2^ represent the cumulative values of all the components of the model. Low and intermediate harvest frequency (red circled), high harvest frequency (blue circled), and low and intermediate frequency samples harvested in April and May (green circled). H-30 days harvesting frequency; I-60 days harvesting frequency; L-90 days harvesting frequency; Jan-a = January, A = April, Mar = March, May = May, Oct = October, Nov = November, Dec = December; Jan-b–January of the next year. R^2^X[1] = 0.472; R^2^Xo[1] = 0.358.

**Figure 3:**
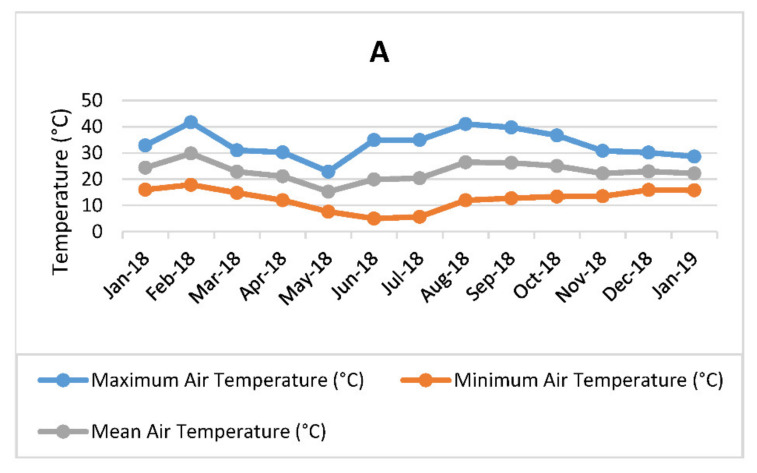

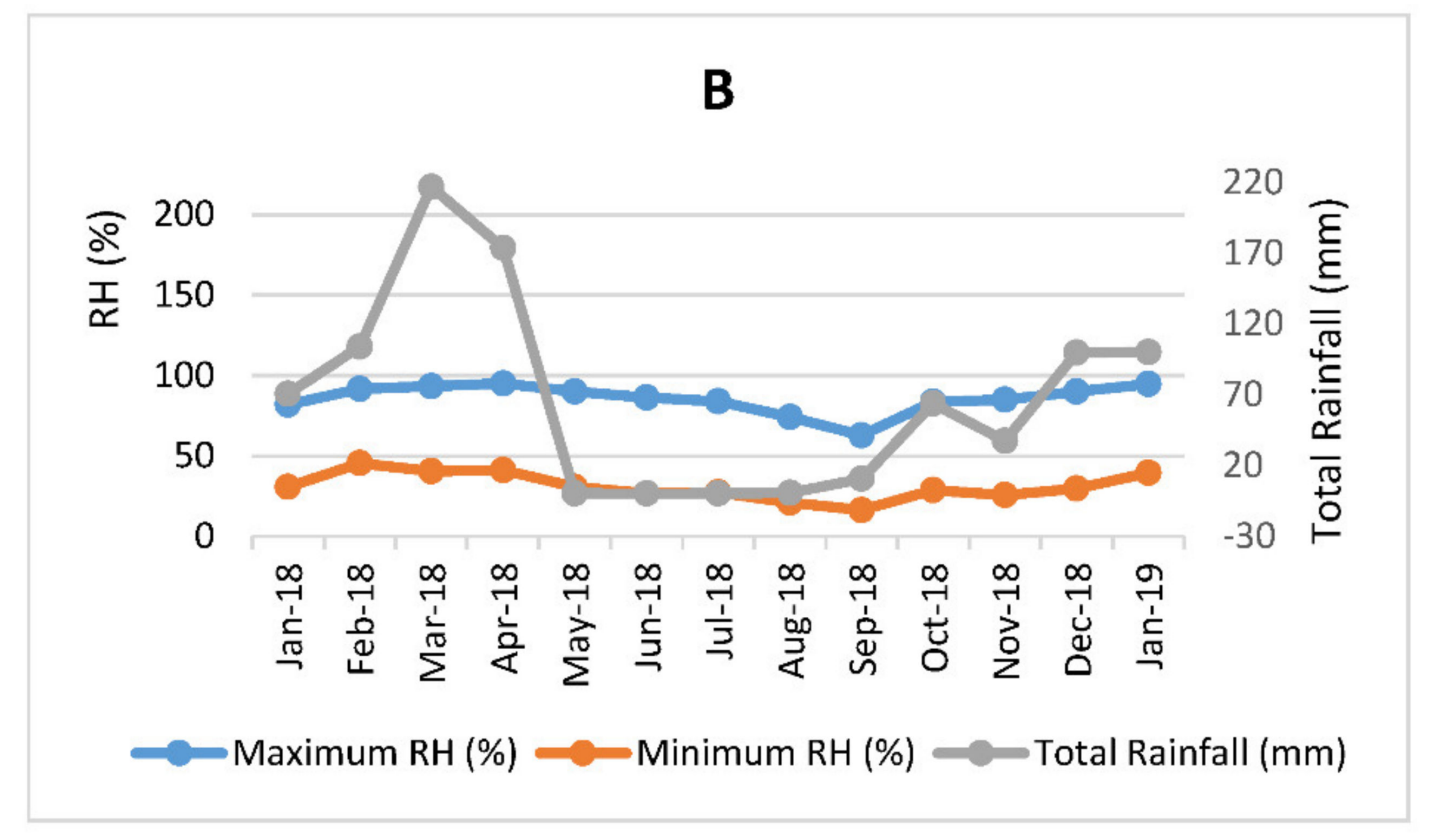
Climatic conditions during the harvesting period-temperature (**A**), relative humidity (RH), and total rainfall (**B**) measured at the University of Pretoria experimental farm in Pretoria, South Africa.

From the spectral data obtained in this study, PCA analysis was conducted and revealed clustering of some samples (Figure 1), which suggest the leaf metabolite profile of *M. oleifera* cultivar in response to different harvesting times and frequencies was affected. The OPLS-DA analysis resulted in clear clustering of the high-frequency samples (Figure 2). However, there was no significant separation between intermediate (60 days) and low (90 days) harvesting frequency treatments, and therefore both treatments could be treated as low harvesting frequency in this study. For March, October, December, and January harvest, the samples from the intermediate and low frequency group (red circled) were separated from the high harvesting frequency group (blue circled) (Figure 2). However, the intermediate and low harvesting frequency samples (green circled) from April and May harvesting were also separated from other intermediate and low harvesting frequency samples (red circled), and this may be associated to the changes in the environmental factors. This was also the same scenario for high frequency sample harvested in March which was selectively placed in between the two major groupings. The temperature shows great variation as it decreased from late March toward the end of May for winter season (Figure 3A). Total rainfall also shows great variation in that period with March and April receiving the highest rainfall (216, 8 mm and 173, 4 mm) during the year (Figure 3B). The effect of environmental factors, especially the effect of temperature and rainfall however needs to be investigated further as samples harvested before and after these months, clustered together and did therefore not show changes in the chemical profile. Given the prevailing seasonal conditions in the area, the period of October to January showed to be a suitable period for evaluating the effect of harvesting on the leaf metabolite profile of the *M. oleifera* cultivar.

Separation of samples as shown by OPLS-DA analysis in Figure 2 indicates that there are certain groups of compounds in *M. oleifera* leaf extract that were affected or responsive to harvesting frequency (red and blue circled) and another group affected by rainfall and temperatures (green circled). The samples collected from April and May, specifically separated from the other samples which is possibly an indication of the changes due to lower temperatures (autumn months) and an unusual high rainfall during this period. The intermediate and low harvesting frequency (60/90 days) seems to be the main determinant for separation of samples of March 2018, October 2018, December 2018, and January 2019 (red circled). Given that the crop management and exposure to environmental conditions were the same for all harvesting treatments, the observed separation of samples between high frequency and low or intermediate samples can certainly be attributed to the effect of harvesting treatments. This is evident for example for samples that were collected in October for the high and low frequency (L-Oct2 vs H-Oct) and January of the following year for high and low frequency treatments (H-Jan2b vs. L-Jan 2b). The low and intermediate samples from April and May harvest (green circled) separated from the other low/intermediate frequency samples (red circled), were grouped closer to the high frequency clustering but also placed far from the high frequency samples that was harvested in the same months.

**Figure 4:**
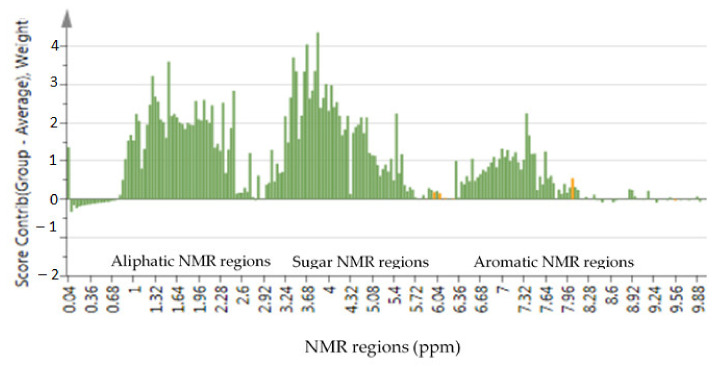
Contribution plot showing metabolites peaks in the NMR aliphatic, sugar, and aromatic regions of *M. oleifera* leaf metabolomics associated with low and intermediate harvesting frequencies clustering (red circled) (Figure 2). Above line, NMR regions were positively associated/contributed to the clustering.

Using the SIMCA software, the contribution plot was constructed, and showed the increase in concentration of most metabolites in aliphatic, sugar, and aromatic regions, which contributed to the separation of the samples (Figure 4), thus also providing key structural information of specific compounds that were detected by ^1^H-NMR spectroscopy. The contribution plot was also used to depict potential marker metabolites associated with changes in the metabolite profile. In terms of the selectively isolated high frequency March sample, the contribution plot (Figure S1) showed both an increase and decrease in particular metabolites peaks, which explain why the sample was not grouped with any of the clusters. In addition to contribution plot, the loading plots were created to show individual scores associated with each analyzed sample (Figure S2). Aromatics in particular, are known to be dominant compounds in plants, and various aromatics such as flavonoids and phenolic glucosides have already been isolated in *M. oleifera* tissues [4,6,26,27]. However, it is confirmed in this study that the accumulation and isolation of these compounds in *M. oleifera* leaf tissues can be irregular, depending on the harvesting frequencies and seasonal changes. Therefore, harvesting practices and environmental conditions could be used to manipulate the production and accumulation of bioactive compounds of *M. oleifera*. This is more practical given that moringa is a tree, and its leaves can be harvested several times within a year.

**Figure 5:**
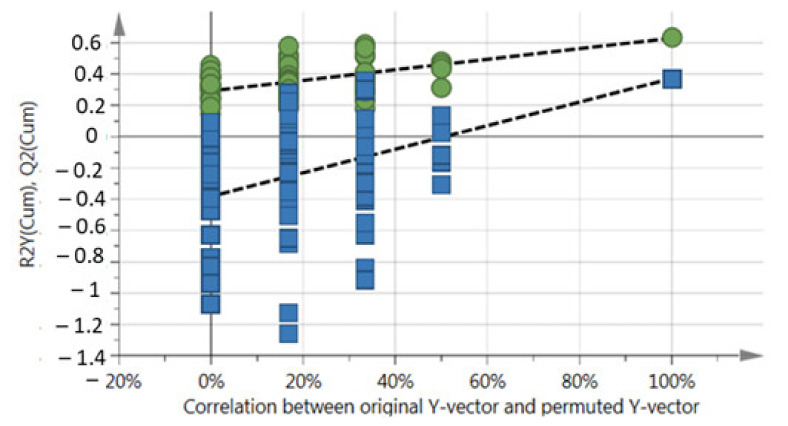
OPLS-DA model validity permutation test for *M. oleifera* (cultivar PKM1) metabolite profile under low, intermediate and high harvesting frequencies. Green circle–R^2^Y; blue square–Q^2^ (R^2^X = 0.832; R^2^Y = 0.643; Q^2^ = 0.371). R^2^ = (0.0; 0.292), Q^2^ = (0.0, −0.381).

**Figure 6:**
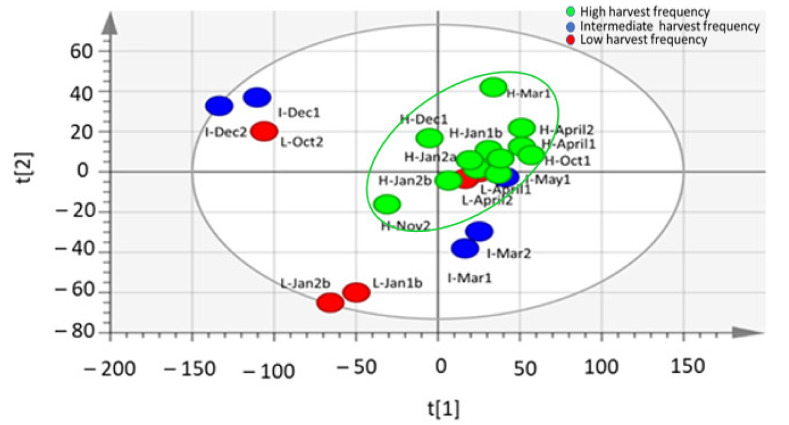
Partial least square (PLS) linear regression score plot showing ^1^H-NMR spectral data of *M. oleifera* (cultivar PKM1) metabolites profile under low, intermediate, and high harvesting frequencies. Low harvest frequency (red dots), intermediate harvest frequency (blue dots), high harvest frequency (green dots). H-30 days harvesting frequency; I-60 days harvesting frequency; L-90 days harvesting frequency; Jan-a = January, A = April, Mar = March, May = May, Oct = October, Nov = November, Dec = December; Jan-b–January of the next year. R^2^X[1] = 0.488; R^2^X[2] = 0.116.

In order to check the validity of the OPLS-DA model, one hundred permutations were performed, and the resulting y-intercept values of R^2^ and Q^2^ were plotted (Figure 5). Although the predictability (Q^2^ = 0.371) of OPLS-DA model was fairly good, further analyses were performed and validated the hypothesis that the harvesting frequency was the main determinant for changes in the metabolite profile. For instance, PLS linear regression analysis was performed to check the time correlation and thus validated that the time factor was not significant as harvesting frequency factor for separation of samples (Figure 6). Once again, all the high frequency samples were grouped together (green circle), without separation on the month of harvest. This was followed by PLS-DA analysis using only the samples from day 0 and only the samples from the endpoint of the time. The predictability of this PLS-DA model was very good (Q^2^ = 0.902), thus confirming the hypothesis, that at time 0 (first harvest) samples are not discriminated but at time end (last harvest) samples were discriminated because of their different harvest histories (Figure S3).

### 2.2. Targeted Metabolites That Were Affected by Changes in the Metabolic Profile

Among phytoconstituents detected in the samples, chlorogenic acid, ferulic acid, vanillic acid, niazirin, wogonin, esculetin, and gamma-aminobutyric acid (GABA) were seven (7) targeted metabolites in this study. These compounds were targeted particularly because they are linked to the changes in the metabolite profile as shown by the contribution plot (Figure 4). In addition, these metabolites are associated with known important medicinal properties, and their presence in *M. oleifera* leaf extract has been widely reported. As indicated in Table 2**,** the targeted compounds were identified using the NMR chemical shifts as previously published [4,6,24,26,28,29,30,31,32], assisted by databases such as Chenomx profiler and the Human Metabolome Database (Supplementary Table S1). Annotation of chlorogenic and GABA were further confirmed through spiking with commercial standards. The fact that most of these bioactive compounds have been isolated in more than one study from *M. oleifera* leaves, also provides support for the annotation of the compounds in this study.

**Figure 7:**
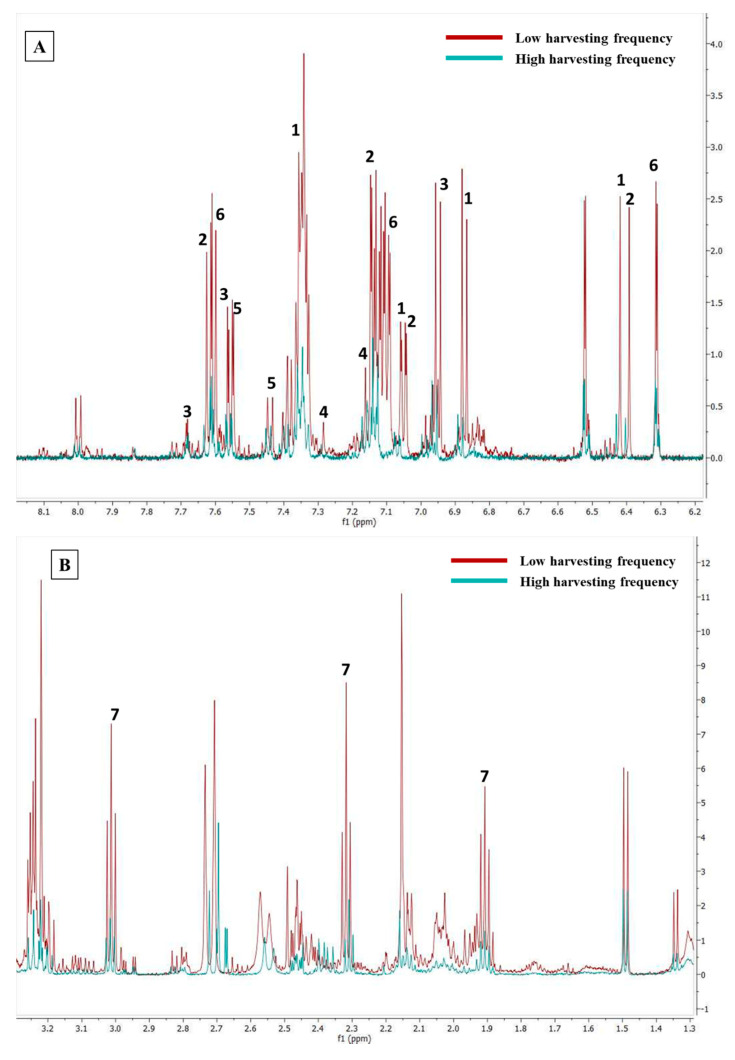
NMR aromatic (**A**) and aliphatic (**B**) region showing difference between high (blue line) and low harvesting (red line) frequency. 1 = Ferulic acid (6.38, 6.86, 7.05, 7.33 ppm); 2 = Chlorogenic acid (6.39, 7.05, 7.15, 7.64 ppm); 3 = Vanillic acid (6.94, 7.55, 7.65 ppm); 4 = Niarizin (7.16, 7.26 ppm); 5 = Esculetin (7.42, 7.55 ppm); 6 = Wogonin (6.32, 7.09, 7.59 ppm); 7 = Gamma-aminobutyric acid (GABA) (1.9, 2.3, 3.0 ppm). Compounds measured in deuterium oxide:deuterated methanol (CD_3_OD) (1:1).

**Figure 8:**
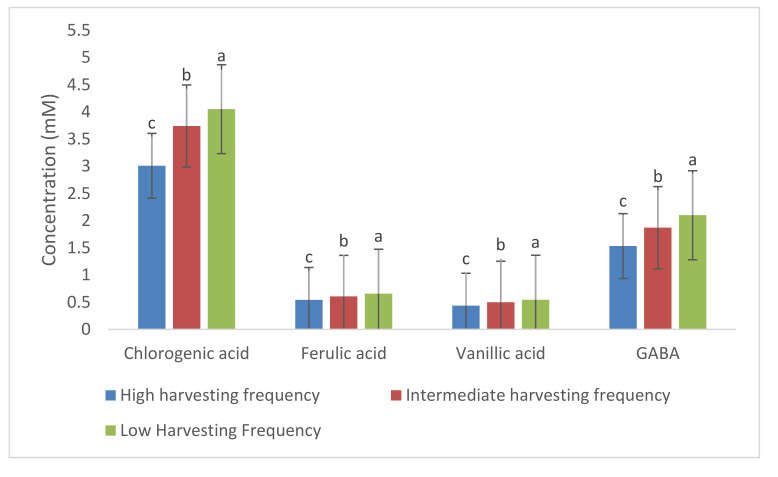
Comparison of mean concentration (mM) of *M. oleifera* leaf chlorogenic acid, ferulic acid, vanillic acid, and gamma-aminobutyric acid (GABA) between low, intermediate and high harvesting frequency plants (*p ≤* 0.05). Different letters (a, b and c) indicate that the mean of three harvesting group were significantly different from each other. *n* = 8 (high harvesting frequency), *n* = 6 (intermediate harvesting frequency), and *n* = 4 (low harvesting frequency).

For each of the compounds, characteristic peaks were used to compare the height of the peaks in different samples, as an indication of the concentration difference. Spectral data (Figure 7A,B) show the increase in concentration level of all these metabolites with low or moderate harvesting frequency as NMR is a quantitative spectroscopic method, where a higher peak represents a higher concentration. This was based on consistent normalization of the TSP peak (0.1%) in all samples. For the plants that were harvested every month (high frequency), the accumulation of these targeted metabolites also remained low in the leaf tissues, which is in accordance with the contribution plot (Figure 4). In addition, there were also other variables with high scores in the contribution plot suggesting that there are other compounds, except the targeted ones, that are affected by harvesting frequency. For chlorogenic acid, ferulic acid, vanillic acid, and GABA, the differences shown in Figure 7A,B were further confirmed by univariate analysis which statistically tested the mean concentration differences among three prescribed harvesting treatments (low, intermediate and high frequency). Various quantitative as well as qualitative differences could be observed, although the focus was on the quantitative differences of the targeted metabolites. Thus, only 4 compounds were quantified, while other compounds were evaluated on the height of the peaks which are indicative of the concentration of the compounds in the samples. The samples used for univariate analysis were those harvested in March 2018, October 2018, December 2018, and January 2019 as they allowed comparison between three prescribed harvesting frequencies. Mean concentration (mM) of integrated peaks for each compound were significantly higher in low and intermediate harvesting frequency plants as compared to high harvesting frequency plants (Figure 8). Therefore, low or moderate harvesting frequency being 60 or 90 days could be used to manipulate the concentration level of some compounds in the leaf of *M. oleifera* cultivars as it appears to be increased in concentration by low and intermediate harvesting frequencies. The 90 days (low) frequency showed the highest mean concentration (Figure 6), but given the demand for continuous supply of the leaves, the 60 days (intermediate) frequency could be the most appropriate harvesting protocol to be standardized. Although direct mechanism to which harvesting frequency can have an impact on the metabolic profile is not well-known, the decrease in concentration of some compounds under high harvesting frequency could be due to probable lower photosynthetic capacity, as the leaves were cut every 30 days. Photosynthesis is a photobiochemical process using light energy to produce adenosine triphosphate (ATP) and nicotinamide adenine dinucleotide phosphate hydrogen (NADPH), which are ultimately consumed in the assembly of carbon atoms in organic molecules [51].

As presented in Table 1, the affected metabolites in this study are associated with various therapeutic activities, and therefore the effect of harvesting frequencies observed in this study is of great interest for the use of *M. oleifera* for medicinal purposes. For instance, the extracts of *M. oleifera* leaves containing niazirin showed inhibitory activity against tumor cells [4]. Presence of chlorogenic, ferulic, and vanillic acid in the *M. oleifera* leaf extract possess potent antioxidant properties, which may be mediated through direct trapping of the free radicals and also through metal chelation [11,28,52,53]. These three phenolic compounds also proved to possess other biological properties, such as anticancer [37,44,54], antimicrobial [40,47], anti-inflammatory [41,42,55], antidiabetic [38,43,56], antispasmodic [37], and reduction of plasma and liver lipids [38,39,57]. Wogonin, esculetin, and GABA also show potential to act as anti-inflammatory, anti-oxidant, and anti-epileptic compounds respectively [33,34,49,50]. This means that there could be valuable gain in considering the harvesting practices in the strategies to optimize bioactive compounds in *M. oleifera* cultivars for the development of plant-based medicinal products. Additionally, the effect of temperature and rainfall is also demonstrated in low frequency harvested samples that showed a similar chemical profile to samples with high harvesting frequency, by clustering together. The study therefore presents support for the effect of harvesting frequency, and potentially other external environmental factors such as rainfall and temperature on the chemical profile of *M. oleifera*.

## 3. Materials and Methods

### 3.1. Hydroponic Planting, Maintenance, and Leaf Harvesting

The experiment was carried out in an open hydroponic system under a shade net (20% white shade cloth) structure at the University of Pretoria experimental farm, Pretoria (S 25° 44′ 53.79″ E 28° 15′ 19.53″). A cheap shade net structure was used instead of polyethylene or polycarbonate structures as the shade cloth covered the greenhouse and needed no expensive cooling systems. This was important given that these types of tunnels are more commonly practiced in small-scale farming systems. The experimental treatments were laid out using a Completely Randomized Design (CRD) with each treatment having twenty (20) replicates. Three harvesting frequency treatments were applied in the study, with the plants harvested at every 30 days (high frequency), 60 days (moderate/intermediate frequency), and 90 days (low frequency) respectively. The trial therefore consisted of 60 individual trees (20 for high frequency, 20 for intermediate frequency, and 20 for low frequency). Each tree was separately harvested and prepared for metabolomic analysis. Sand and coir mixture at the ratio of 3:1 were used for planting the seeds of *M. oleifera* PKM1 cultivar in 20 L nursery plastic bags. Hydroponic fertilizer (Hygroponic + Solu-Cal, Hygrotech, Pretoria) was applied through drip irrigation (fertigation). Irrigation was scheduled twice in a day, with each plant provided with 666 mL of water in the morning and 550 mL in the late afternoon. With the open hydroponic system, the irrigation supplements the annual rainfall, as water did penetrate the shade net structure. Other essential crop management practices were equally applied throughout the experimental period. The climatic conditions during the period of harvest was also recorded (Figure 3). The material was propagated by seed and eight months after planting the seeds, first leaf harvesting started in January 2018 and continued to January of the next year. The leaf batches were collected each time from the same plants, harvesting at 50% severity to allow enough leaves to be collected in the next harvest—especially for 30 days harvesting frequency. The middle-plant mature leaves (single leaflets) were collected at each harvesting period. For all the harvesting treatments, the leaf samples were collected on the same date of each harvest month and in the morning. The leaf samples from each treatment were air-dried at room temperature in the dark, ground in a blender into fine powder and sieved through a 2 mm sieve, and stored in the −80 °C freezer until further analyses.

### 3.2. Preparation of Extract for Metabolomics Analysis

Powdered leaf material of 50 mg per sample was weighed and stored in the 2 mL Eppendorf tubes for direct extraction of metabolites. To the sample, 0.75 mL of deuterated methanol (CD_3_OD) and 0.75 mL of potassium dihydrogen phosphate (KH_2_PO_4_) were added and buffered in deuterium water (D_2_O) (pH 6.38) containing 0.1% (*w*/*w*) TSP (Trimethylsilylpropionic acid sodium salt) (Sigma-Aldrich, Modderfontein, South Africa). In order to mix the reagents, the samples were vortexed at room temperature for 1 min. The mixture was then ultrasonicated for 15 min to break down the cell walls after which it was centrifuged for 20 min to separate the supernatant from the pellet. The supernatant from each tube was then transferred to a 5 mm NMR tube for analysis.

### 3.3. Data Acquisition, Processing, and ^1^H-NMR Spectra Analysis

^1^H-NMR was used for data acquisition using 600 MHz NMR spectrometer (Varian Inc., California, CA, USA). Spectra were recorded on a 28-shim Varian VNMRS (DDR-1) Premium Shield system operating at a nominal proton frequency of 600.13 MHz. The system is equipped with a dedicated 5 mm H{CN} triple resonance (indirect detection) room temperature probe head attached to a pulsed field gradient generator, an Agilent 7510-AS 12-way autosampler and associated CPU running VNMR 4.2A software. All spectra are acquired using standard PROTON parameters under automated locking/shimming/acquisition conditions at a standard temperature of 30 °C, 14 ppm sweep width, 45° pulse angle, and no recycle delay. No solvent suppression was applied and the chemical shift range δ 3.23–3.36 ppm representing residual methanol, as well as the chemical shift range δ 4.6–5 ppm representing water [58], were excluded from further analysis. MestReNova software (version 12.0.4, Mestrelab Research, Santiago de Compostela, Spain) was used for the pre-processing of the spectral data. Normalization and baseline correction was consistently applied to all sample spectra as well as calibration to an internal standard TSP of 0.0 ppm. The spectral intensities were reduced to integrated regions, also referred to as buckets or bins, of equal width (0.04 ppm each) corresponding to the region of 0.04–10.00 ppm. Multivariate data analysis by means of principal component analysis (PCA), partial least square discriminatory analysis (PLS-DA), and orthogonal partial least square discriminatory analysis (OPLS-DA) were conducted using SIMCA software (SIMCA version 15.0.2, Umetrics, Umeå, Sweden) applying pareto scaling. To check for time factor correlation, PLS liner regression model was performed by applying the observation ID (harvesting frequencies) as a predicted Y-variables and primary variable ID (NMR spectral integrals, intensities) as a predictor X-variables.

Absolute quantification analysis was then used to identify the changes in the chemical profile using the NMR chemical shifts of the targeted compounds (chlorogenic acid, ferulic acid, vanillic acid, esculetin, wogonin, niazirin, and GABA) as previously published, assisted by databases such as Chenomx profiler and the Human Metabolome Database (Table S1). For GABA and chlorogenic acid, spiking with commercial chemicals was also done to confirm their annotation. Spiking was limited to these two compounds, and the other compound peaks were only annotated. According to the metabolite identification levels defined by [51], spiked compounds fall to identification level 3 while the compounds that putatively annotated without chemical reference standards but based upon physicochemical properties and/or spectral similarity with public/commercial spectral libraries fall to identification level 2. Furthermore, mean concentration (mM) for chlorogenic acid, ferulic acid, vanillic acid, and GABA was determined using Chenomx–NMR suite 8.4 (Chenomx Inc, Edmonton, AB, Canada). The concentrations of these compounds were calculated based on the integration values of the peaks in each molecule and compared to the normalized TSP peak (0.1%). The peaks were aligned with the sample peaks in Chenomx to ensure that the correct molecules were quantified. This allowed computation of univariate analysis for each compound using one-way analysis of variance (ANOVA), which supplemented visual comparison of spectral data. The ANOVA analysis was followed by Fisher’s Least Significant Difference (LSD) test to statistically compare the mean of one group with the mean of another group. The concentrations presented, therefore provides the concentration of each compound in the leaves of the sample.

## 4. Conclusions

The findings of this study provide insight into the effect of harvesting frequencies on the metabolite profile and associated medicinal activity of the *M. oleifera* leaf materials. Clustering of samples is primarily based on harvesting frequency, although environmental factors are proposed to be affected by the clustering of the low and intermediate harvesting frequency samples collected in April and May as the samples before and after these months clustered together. The separation of these samples from the main cluster is therefore proposed to be linked to the high rainfall which was measured during this time and the effect of rainfall should therefore be confirmed in future studies. Low and intermediate harvesting frequency increased the concentration level of most compounds, including compounds such as esculetin, wogonin, niazirin, chlorogenic acid, ferulic acid, vanillic acid, and GABA. The study also shows the concentration differences of chlorogenic acid, ferulic acid, vanillic acid, and GABA, as a result of high, intermediate, and low harvesting frequency treatments. The current study also broadens the understanding of how Moringa chemical composition could be varied under specific growing and harvesting conditions. Therefore, the interpretation of Moringa qualities should not be generalized, but also take into the consideration how the plants were cultivated, harvested, and processed. Overall, this study suggests that there is a need to precisely develop production systems and follow standardized harvesting protocols to optimize the concentration of active compounds in the *M. oleifera* leaf extracts. Lastly, it is recommended for future studies to investigate affected metabolic pathways in order to establish mechanism to which harvesting frequency can have an impact on the leaf metabolite profile of the Moringa tree.

## Figures and Tables

**Table 1:** Sampling scheme for *M. oleifera* leaves extracts used for NMR metabolomics analysis.

Harvest Date	Harvest Frequency	Sample Labels
January 2018	High	H-Jan1a H-Jan2a H-Jan3a
	Intermediate	I-Jan1a * I-Jan2a *
	Low	L-Jan1a * L-Jan2a *
February 2018	High	H-Feb1 ** H-Feb2 **
March 2018	High	H-Mar1 H-Mar2 **
	Intermediate	I-Mar1 I-Mar2
April 2018	High	H-April1 H-April2
	Low	L-April1 L-Apri2
May 2018	High	H-May1 H-May2
	Intermediate	I-May1 I-May2 **
June 2018	Excluded from metabolomics analysis as the leaf quality was affected by the winter season	
July 2018		
August 2018		
September 2018		
October 2018	High	H-Oct1 H-Oct2
	Intermediate	I-Oct1 ** I-Oct2 **
	Low	L-Oct1 ** L-Oct2
November 2018	High	H-Nov1 H-Nov2
December 2018	High	H-Dec1 H-Dec2 **
	Intermediate	I-Dec1 I-Dec2
January 2019	High	H-Jan1b H-Jan2b
	Low	L-Jan1b L-Jan2b

* Excluded from analysis as day zero. ** Removed from the analysis as the outliers.

**Table 2:** Targeted metabolites that were annotated in the ^1^H-NMR fingerprints. The NMR values obtained and the medicinal properties for each compound are presented.

Compounds Name	Chemical Shift Peaks (ppm)	Identification Level *	Associated Medicinal Properties	References
Niazirin	δ 7.16, 7.26	Level 2	Anticancer activities	[4,6]
Wogonin	δ 6.32, 7.09, 7.59	Level 2	Anti-inflammatory	[33]
Esculetin	δ 7.42,7.55	Level 2	Antioxidant, anti-inflammatory	[34]
Chlorogenic acid	δ 6.410, 7.05, 7.15, 7.64	Level 3	Anti-inflammatory, anti-oxidants, antispasmodic, anti-cancer, anti-obesity	[35,36,37,38,39]
Ferulic acid	δ 6.38, 6.86, 7.05, 7.33	Level 2	Anti-oxidants, anti-microbial, anti-inflammatory, anti-diabetic	[28,40,41,42,43,44]
Vanillic acid	δ 6.94, 7.55, 7.65	Level 2	Anti-inflammatory, anti-oxidants, anti-cancer, anti-sickling, anti-microbial	[36,45,46,47]
Gamma-aminobutyric Acid (GABA)	δ 1.9, 2.3, 3.0	Level 3	Anti-epileptic, anti-anxiety	[48,49]

* Level 2 = putatively annotated compounds without chemical reference standards, but based upon physicochemical properties and/or spectral similarity with public/commercial spectral libraries; Level 3 = spiking with commercial chemicals to confirm their annotation [50].

## Data Availability

The data for the study was deposited in a publicly available database: Prinsloo, Gerhard (2020), “Moringa hydroponics”, Mendeley Data, V1, doi:10.17632/dvzrggnjn9.1. It can be accessed at: http://dx.doi.org/10.17632/dvzrggnjn9.1.

## Supplementary Materials

The following are available online, Figure S1: Contribution plot showing metabolite peaks in the NMR aliphatic, sugar and aromatic regions of M. oleifera leaf metabolomics associated with March sample separation under high harvesting frequency as shown in Figure 2, Figure S2: OPLS-DA loading score plot showing 1H-NMR spectral data of M. oleifera (cultivar PKM1) metabolite profile under low/ intermediate and high harvesting frequency, Figure S3: PLS-DA score plot showing 1H-NMR spectral data of M. oleifera (cultivar PKM1) metabolite profile under low/intermediate and high harvesting frequencies, showing separation between day 0 and end point harvesting, Figure S4: PLS-DA model validity permutation test for M. oleifera (cultivar PKM1) metabolite profile under low, intermediate and high harvesting frequencies, Table S1: 1H-NMR spectral regions of annotated compounds that contributed to the separation of leave samples.

## Sample Availability

Metabolomics data associated to this manuscript is available in a publicly available database, Mendeley Data, http://dx.doi.org/10.17632/dvzrggnjn9.1.
