# Supplementary material for: NMR-Based Metabolomic Analyses to Identify the Effect of Harvesting Frequencies on the Leaf Metabolite Profile of a Moringa oleifera Cultivar Grown in an Open Hydroponic System
Source: Molecules. 2021 Apr 15;26(8):2298. doi: 10.3390/molecules26082298 (PMC8071497; doi:10.3390/molecules26082298)
Supplement: Supplementary file 1 [file molecules-26-02298-s001.zip › molecules-1037468-supplementary.pdf]

Supplementary Materials

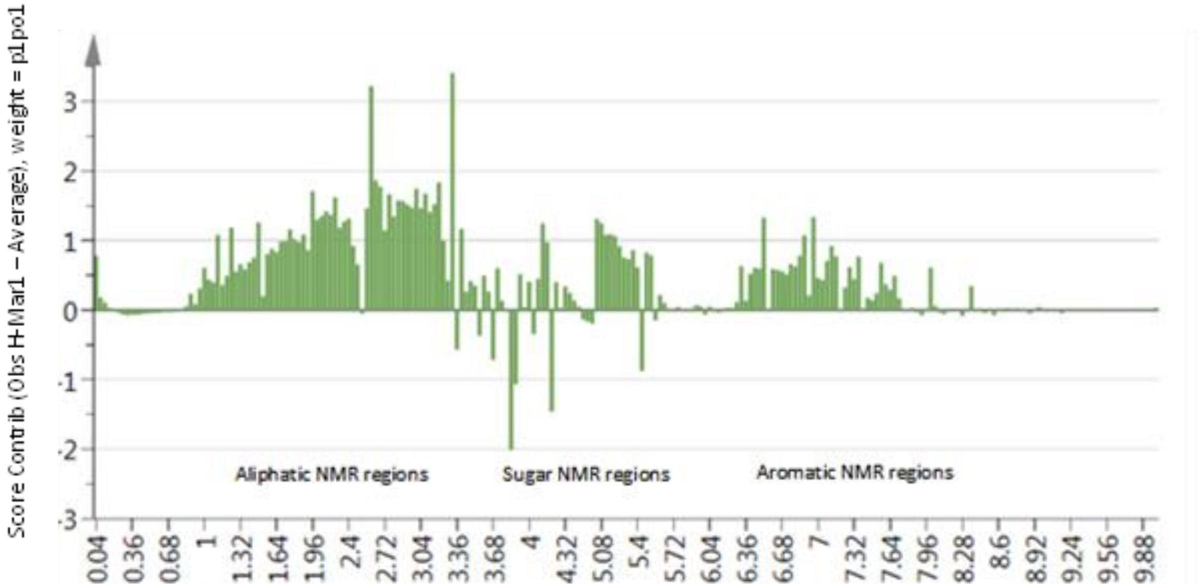

NMR regions (ppm)

Figure S1. Contribution plot showing metabolites peaks in the NMR aliphatic, sugar and aromatic regions of *M. oleifera* leaf metabolomics associated with March sample separation under high harvesting frequency as shown in Figure 2.

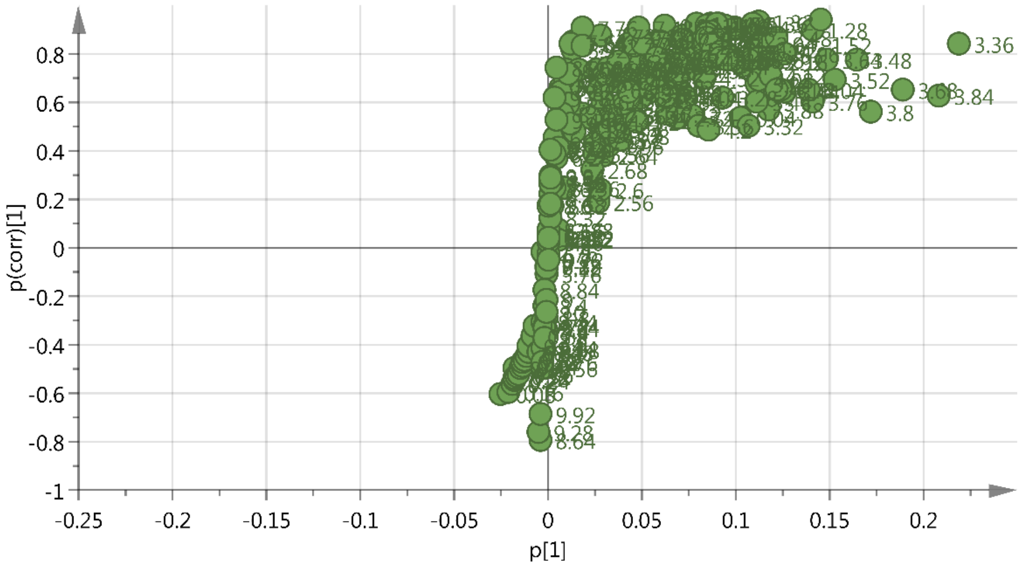

Figure S2. OPLS-DA loading score plot showing ^1H NMR spectral data of *M. oleifera* (cultivar PKM1) metabolite profile under low/ intermediate and high harvesting frequency ($R^2X[1] = 0.472$; $R^2Xo[1] = 0.358$)

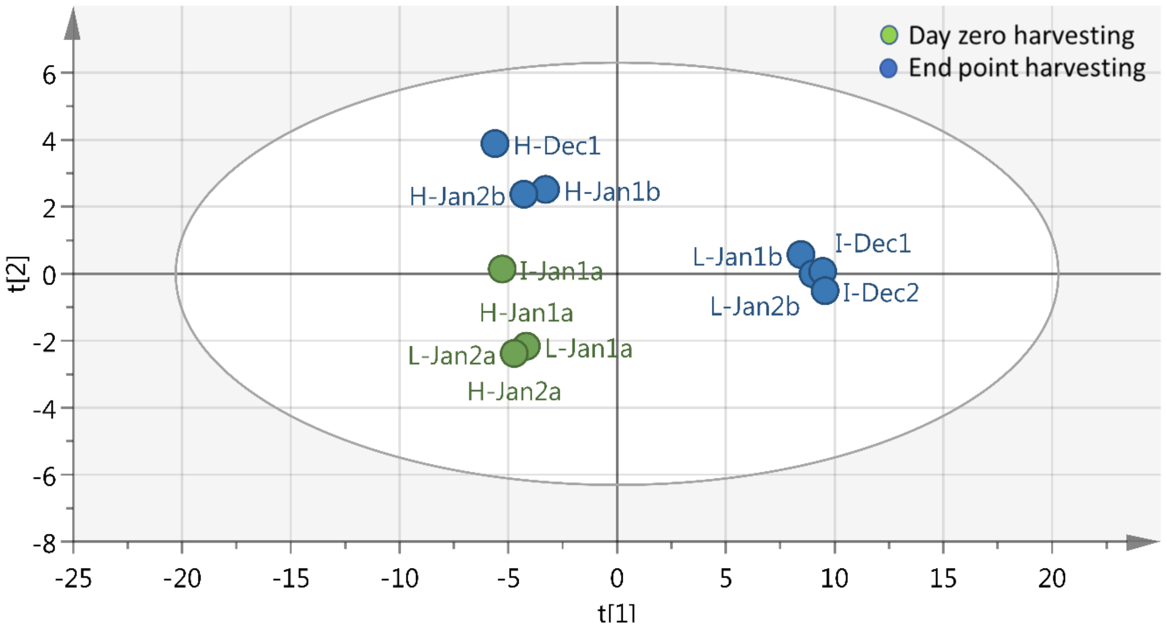

Figure S3. PLS-DA score plot showing ^{1}H NMR spectral data of *M. oleifera* (cultivar PKM1) metabolite profile under low/ intermediate and high harvesting frequencies, showing separation between day 0 and end point harvesting (R^2X = 0.954; R^2Y = 0.93; Q^2 = 0.902). H-Jan = high frequency; I-Jan = intermediate frequency; L-Jan = low frequency. $R^2X[1]$ = 0.88; $R^2X[2]$ = 0.0746

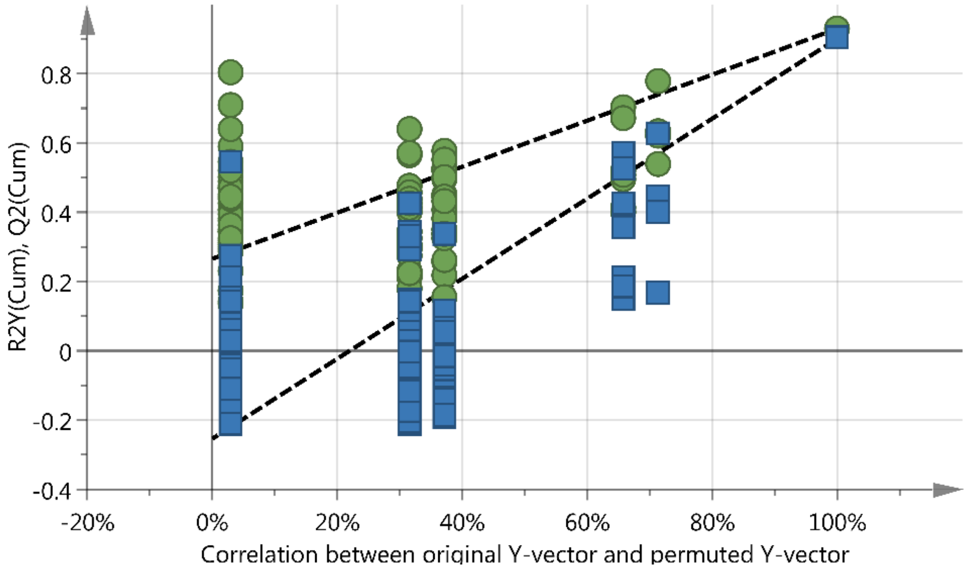

Figure S4. PLS-DA model validity permutation test for *M. oleifera* (cultivar PKM1) metabolite profile under low, intermediate and high harvesting frequencies. (R^2X = 0.954; R^2Y = 0.93; Q^2 = 0.902). R^2 = (0.0; 0.266), Q^2 = (0.0, - 0.254)

Table S1 ^{1}H-NMR spectral regions of annotated compounds that contributed to the separation of leave samples.

**Treatment**	**Compounds**	**^{1}H-NMR Chemical shifts (ppm)**	**Referenced chemical shifts (ppm)**	**References**	**Chenomx (ppm)**	**Assigned numbers**
Low and intermediate harvesting frequency	Ferulic acid	6.38, 6.86, 7.05 7.33	6.388, 6.86, 7.05, 7.3	(Anselmi et al., 2006) https://hmdb.ca/spectra/nmr_one_d/1934 (HMDB accessed 16.08.2020)	6.4, 6.9, 7.1	1
	Chlorogenic acid	2.02, 2.17, 6.39, 7.05, 7.15, 7.64	2.02, 2.18, 6.39 7.05, 7.14, 7.63	https://hmdb.ca/spectra/nmr_one_d/1934 (HMDB accessed 16.08.2020) (Sabino et al., 2019)	6.4, 6.9, 7.1 7.6	2
	Vanillic acid	6.94, 7.55, 7.65	6.93, 7.54, 7.64	https://hmdb.ca/spectra/nmr_one_d/1390 (HMDB accessed 16.08.2020) (Sakushima et al., 2003)	6.9, 7.4, 7.5	3
	Niazirin	1.2, 3.84, 7.16, 7.26	1.21, 3.81, 7.2, 7.26	(Faizi et al., 1994; Gilani et al., 1994; Maurya et al., 2011)		4
	Esculetin	3.21, 3.35, 7.42, 7.55	3.21, 3.35, 7.4 7.5	(Aboul-Ela et al., 2002)		5
	Wogonin	3.86, 6.3, 7.09, 7.59	3.86, 6.32, 7.09 7.67	(Jang et al., 2005; Li et al., 2009)		6
	GABA	1.9, 2.3, 3.01	1.88, 2.28, 3.01	(Kaiser et al., 2008)	1.9, 2.3, 3.0	7
